# Case Report: Good cardiac tolerance to Toripalimab in a CVD patient with oral melanoma

**DOI:** 10.3389/fphar.2022.890546

**Published:** 2022-08-01

**Authors:** Wei Pan, Li Yin, Yadi Guo, Dachao Pan, Hui Huang

**Affiliations:** ^1^ Cardiovascular Department, The Eighth Affiliated Hospital, Sun Yat-Sen University, Shenzhen, China; ^2^ Oncology Department, The Eighth Affiliated Hospital, Sun Yat-Sen University, Shenzhen, China

**Keywords:** melanoma, immunotherapy, cancer treatment, arrhythmic mitral valve prolapse, cardiovascular toxicity

## Abstract

Primary oral melanoma is extremely rare, and the prognosis is very poor. With the development of immunotherapy, melanoma’s treatment landscape changed dramatically. Toripalimab, a recombinant programmed death receptor 1 (PD-1) monoclonal antibody, has been approved as second-line therapy for metastatic melanoma. However, the cardiac toxicity of Toripalimab is seldom reported. This article describes the application of Toripalimab on a patient who suffered from primary oral melanoma accompanied with arrhythmic mitral valve prolapse (AMVP).

**Case Summary:** A 55-year-old Chinese female was diagnosed with BRAF wild-type oral malignant melanoma by excisional biopsy and genetic test. The melanoma quickly progressed after complete tumor resection. Combined therapy after surgical resection was applied to control the progression of melanoma. Due to this patient’s basic cardiovascular situation, sacubitril–valsartan, spironolactone, and bisoprolol were used to maintain cardiac function. After five antitumor treatment courses, we re-evaluated the patient systemically from the symptom, physical examination, and auxiliary examination. The result showed that the patient who received Toripalimab combined with chemotherapy and radiotherapy did not present severe side effects on the cardiovascular system. The cardiac function remained well.

**Conclusions:** This case provided evidence of Toripalimab combined with chemotherapy on melanoma patients with complex cardiovascular diseases. Toripalimab demonstrated a manageable safety profile and durable clinical response. In addition, the standard CHF treatment plays a vital role in the protection of cardiac function. In a cancer patient with complex cardiovascular diseases, standard prophylactic CHF treatment should be applied at an early stage.

## Introduction

Melanoma is a severe malignant tumor derived from the skin and mucous membrane with its aggressive behavior and less favorable prognosis (Moţăţăianu et al., 2019). The epidemiological data showed that the incidence of this cancer elevated rapidly globally, and the overall 5-year survival rate for mucosal melanomas is 0%–45% (Yde et al., 2018). However, the management of melanoma patients is a complex and evolving issue. Recent data indicate that immunotherapy may improve this situation in the years to come. Anti-CTLA-4 and anti-PD-1/PD-L1 blockade in mucosal melanoma have been evaluated in recent studies. PD-1 monoclonal antibody is considered as a safe, feasible, and effective therapy for patients with metastatic melanoma (Carreau and Pavlick, 2019; Goldberg et al., 2020). However, some cardiovascular events such as myocarditis, myocardial infarction, pericardial effusion, pericarditis, arrhythmia, cardiac tamponade, and acute heart failure have been recently reported (Läubli et al., 2015; Moreira et al., 2019). Though the cardiovascular side effects are relatively rare, they can be severe and potentially fatal when they develop. So a close collaboration between cardiologists and oncologists is needed to help cancer patients receiving the PD-1 antibody, especially when treating patients with basic cardiovascular disease. Toripalimab is one of the promising anti-PD-1 antibodies, which was warranted by its efficacy and safety in the treatment of melanoma (Tang et al., 2020a). Nevertheless, the cardiac tolerance of Toripalimab is still unknown because of insufficient evidence. Here, we reported a case of metastatic melanoma with Toripalimab treatment combined with chemotherapy, who suffered from long-lasting heart valve disease and arrhythmia. Under standard management of cardiovascular diseases, the patient presented good cardiac tolerance in the antitumor treatment of melanoma.

## Case description

**FIGURE 1:**
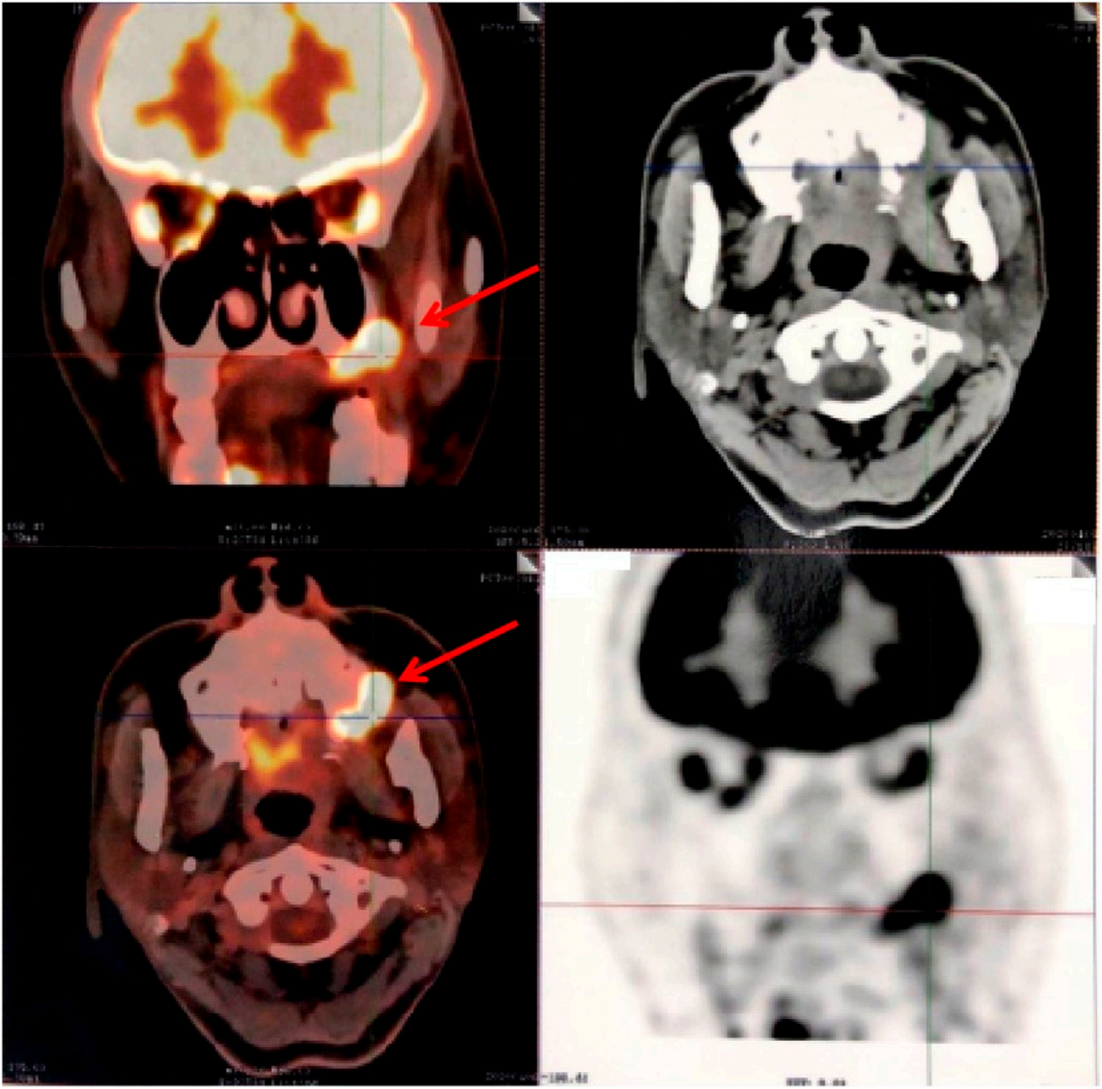
PET-CT axial view shows metabolic hyperactivity of the maxillary mass on the left lateral wall.

**FIGURE 2:**
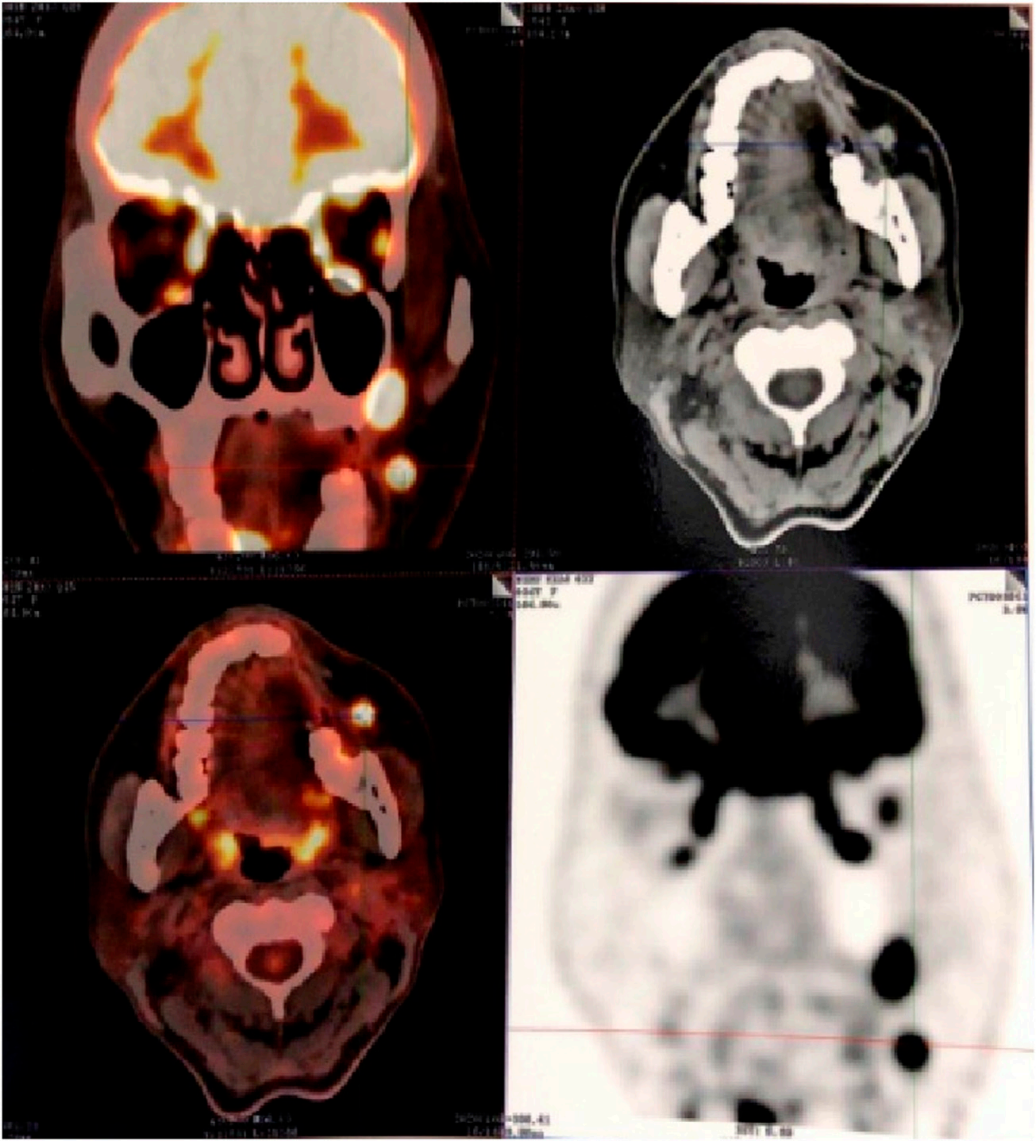
PET-CT axial view shows metabolic hyperactivity of the lymph node mass on the left side.

**FIGURE 3:**
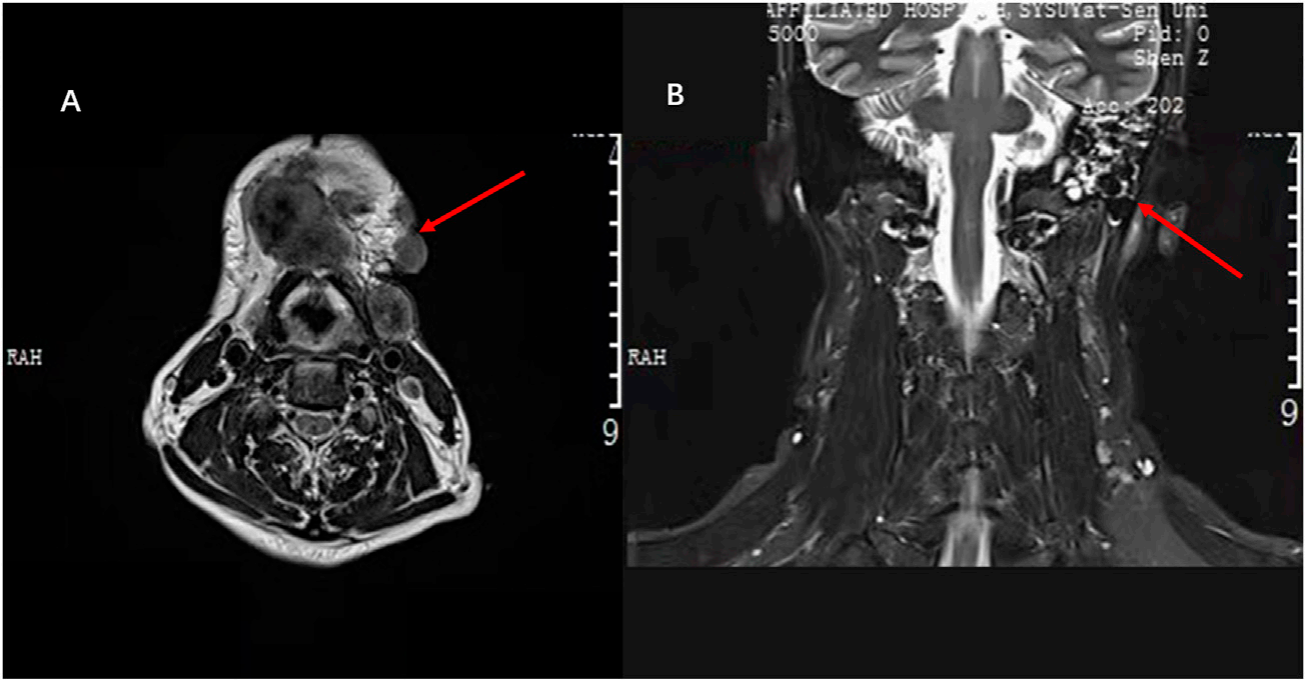
MRI image of metastatic melanoma. **(A)** T2-weighted axial image shows soft tissue mass with ill-defined borders involving the left lateral wall and cervical lymph node metastasis. The lesion is causing compression of oropharynx. **(B)** Post contrast T2-weighted coronal image shows the enlarged cervical lymph node.

**FIGURE 4:**
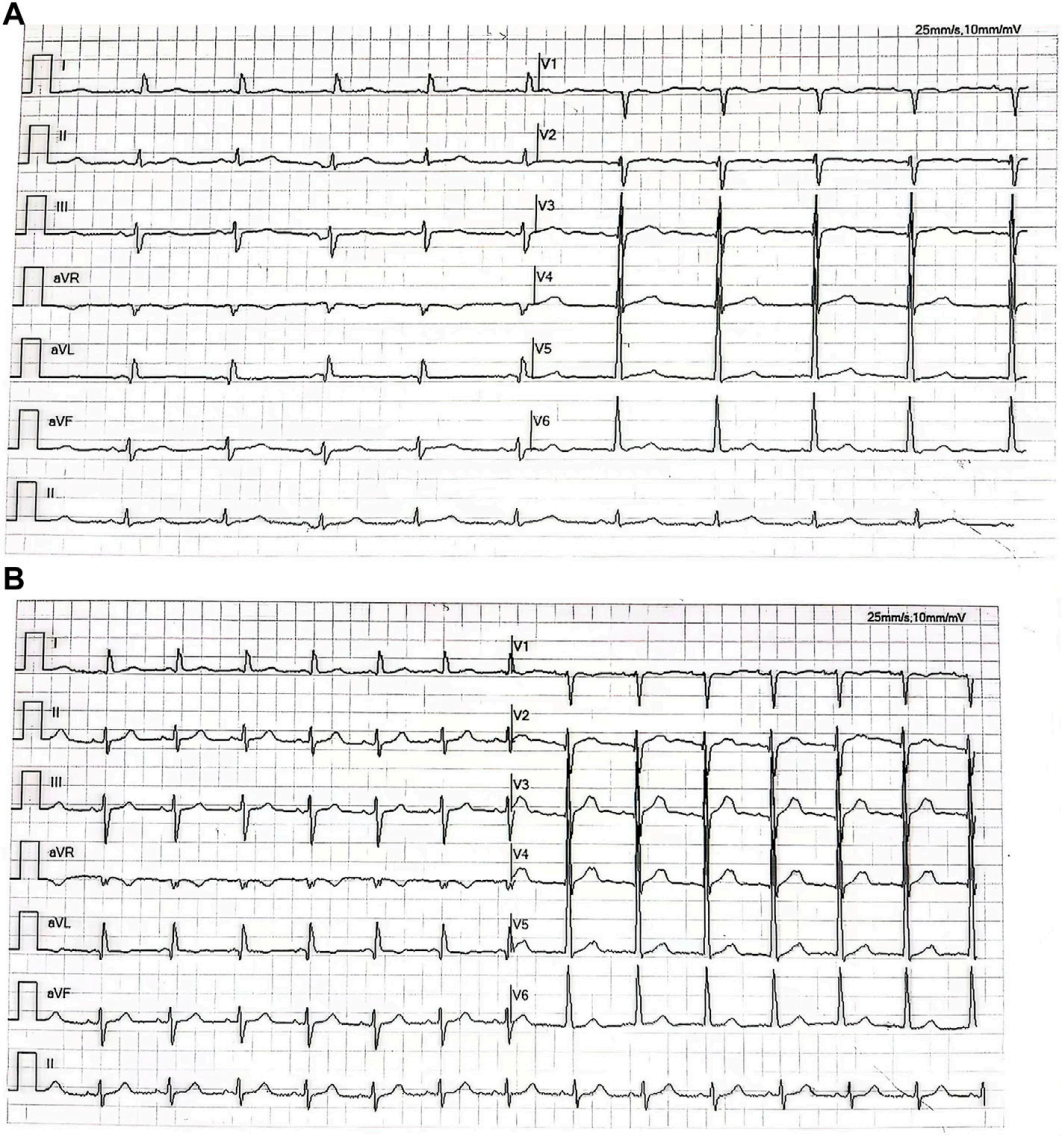
**(A)** Electrocardiogram shows the sinus rhythm with high voltage on the V5 lead before the antitumor therapy. **(B)** Electrocardiogram shows sinus rhythm with high voltage on the V5 lead after the five-course antitumor therapy.

**FIGURE 5:**
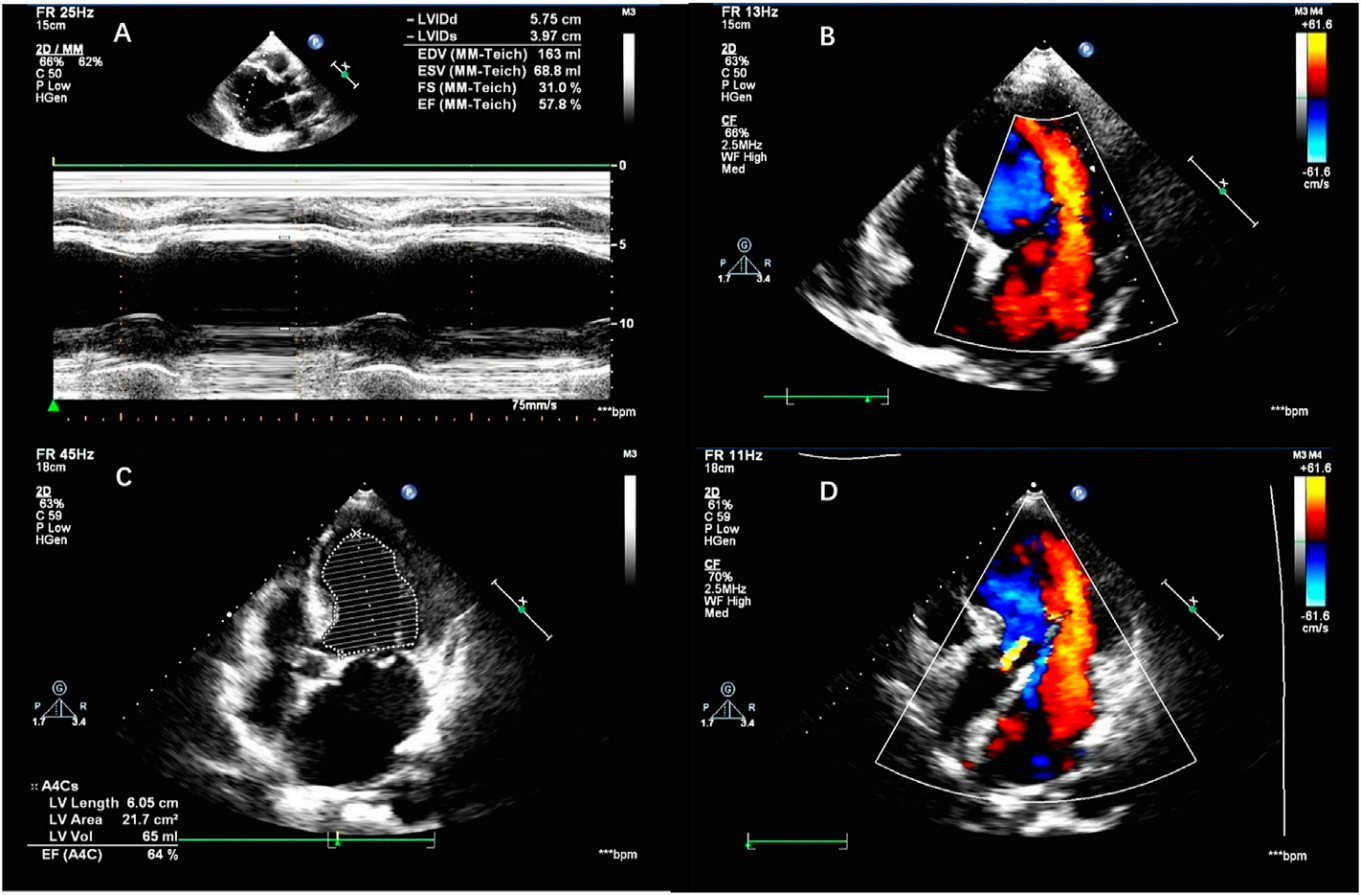
**(A,B)** Echocardiography before the antitumor therapy. **(C,D)** Echocardiography after the five-course antitumor therapy.

A 55-year-old woman was admitted to the hospital in December 2019, with a chief complaint of repeated gingival bleeding for 2 months. She also had a history of mitral prolapse and mitral regurgitation, as well as a history of premature ventricular contraction. Before this admission, she received a standard prophylactic therapy including sacubitril–valsartan, spironolactone, and bisoprolol to prevent heart failure. There are no symptoms of chest stuffiness, chest pain, dyspnea, etc. Exercise tolerance is well maintained with a preserved left ventricular ejection fraction (LVEF). Her heart rate was well controlled in a range of 60–70 beats/min. A 24-h Holter electrocardiogram (ECG) at baseline showed sinus rhythm with an average heart rate of 53 bpm. There were 11,619 isolated ventricular ectopic beats (VEBs), and a total of 26 nonsustained ventricular tachycardia events were registered. A rapidly growing mass at the left posterior edentulous maxillary alveolar ridge was found and excised for histopathology analysis after the careful oral examination. The biopsy specimen revealed a sheet of pleomorphic epithelioid tumor cells interspersed with melanophages with melanin pigmentation. Then the tumor cells were stained positive for human melanoma black (HMB)-45, melan-A, and S-100 protein by immunohistochemical analysis, which concluded the diagnosis of malignant melanoma. Computed tomography results revealed the destruction of the maxillary bone and the formation of soft tissue mass–like lesions. Therefore, complete removal of the tumor, including the mass of the parotid gland as well as the left lateral neck dissection, was operated quickly for neoplasm staging and the treatment of melanoma. Further molecular analysis and positron emission tomography/computed tomography (PET/CT) showed that this melanoma was a stage IIIc, pT4N3M0, BRAF wild-type melanoma with the lymph node metastasis (Figure 1). Though there was no clinical or radiologic evidence of the liver, bone, cerebral, or pulmonary metastasis at the moment (Figures 1, 2), the surgical therapy seemed to fail because of the rapid progress of melanoma in the lymph node (Figure 3). Due to this reason, the strategy of chemotherapy combined with immunotherapy was initiated. Temozolomide combined with Toripalimab was chosen for this patient. Considering that the patient suffered from basic cardiovascular diseases, biomarkers such as cardiac troponin I (cTnI) and N-terminal pro B-type natriuretic peptide (NT-proBNP) were detected regularly during the antitumor course (Table 1). ECG (Figure 4) and ultrasonic cardiogram (UCG) (Figure 5) were also performed to evaluate the cardiac function. A 24-h Holter ECG after immunotherapy showed sinus rhythm with an average heart rate of 49 bpm. There were 524 isolated VEBs. The treatment for the prevention of HF was continually conferred during the five courses of antitumor therapy with temozolomide and Toripalimab. The total time of five-course combined therapy was more than 6 months, and there were no cardiovascular events such as myocarditis, myocardial infarction, pericardial effusion, pericarditis, arrhythmia, or occurrence of cardiac tamponade. The aforementioned cardiovascular indexes evaluated that there were no abnormal changes observed in this female patient. It showed good cardiac tolerance to Toripalimab in combination with chemotherapy under the protection of cardiovascular regimen.

**TABLE 1:** Levels of myocardial enzymes and NT-proBNP before and after immunotherapy in the patient.

Courses after immunotherapy)	Myo (ng/ml)	CK-MB(ng/ml)	cTnI (ng/ml)	NT-proBNP(pg/ml)
0	27.1	0.47	<0.012	173
1	27.6	0.69	<0.012	157
2	21.2	0.35	<0.012	138
3	25.1	0.54	<0.012	229
4	25.9	0.59	<0.012	165
5	21.5	0.3	<0.012	188

## Discussion

Metastatic melanoma, especially derived from primary oral melanoma, is a rare and aggressive category of cancer with a high mortality rate and a poor prognosis (Moţăţăianu et al., 2019). Treatment with ICIs has improved clinical outcomes in multiple types of cancer (Yoneda et al., 2018; Shi et al., 2020; Keenan and Tolaney, 2020), especially in the treatment of melanoma (Robert et al., 2020), but the combined effects of immunotherapy and chemotherapy have not been sufficiently studied. In addition, as the mechanism of immunotherapy is to recognize and target cancer cells by attacking the immune system directly, it may also cause damages to the normal tissues. Previous studies found that unlike most immune-related adverse events, which are common, reversible, and can be treated effectively with glucocorticoid therapy, cardiovascular side effects are usually uncommon but with severe consequences and sometimes may lead to sudden death. Cardiotoxic side effects such as autoimmune myocarditis, pericarditis, and vasculitis have been reported in patients interfering with the CTLA-4 and PD-1 axes (Weyand et al., 2018; Spisarová, 2020; Lal et al., 2021; Fu et al., 2020). Therefore, side effects caused by ICIs have begun to attract widespread concerns.

As we all know, therapeutic antibodies against programmed death receptor 1 (PD-1) are considered to be efficient and safe therapy in metastatic melanoma. Theoretically, interference with the CTLA-4 and PD-1 axes can lead to serious and potentially fatal cardiovascular toxicity. The POLARIS-01 multicenter phase II trial was designed to evaluate safety and efficacy of Toripalimab in advanced Chinese patients with melanoma who had failed in systemic treatments. The result showed that patients with positive PD-L1 staining in tumor biopsies had significantly improved ORR (38.5% vs. 11.9%, *p* = 0.0065), PFS (7.7 months vs. 2.7 months, *p* = 0.013), and OS (not reached vs. 14.4 months, *p* = 0.0005) than PD-L1-negative patients which confirmed the safety of Toripalimab (Tang et al., 2020). However, cardiovascular toxicity is rarely reported in Toripalimab, not to mention the situation when treated with PD-1 together with chemotherapy in metastatic melanoma patients with basic cardiovascular diseases. In this case report, the female patient diagnosed with metastatic melanoma also suffered from AMVP. AMVP was defined as the disease of mitral valve prolapse (MVP) with ventricular arrhythmias (VAs) on Holter monitoring, including inverted or biphasic T waves, QT dispersion, QT prolongation, and premature ventricular contractions originating from the left ventricular out flow tract and papillary muscles (Essayagh et al., 2020). From ECG, we could read the situation of this patient who satisfies the diagnostic standard of AMVP. Recent RCTs suggested that sudden cardiac death (SCD) could occur earlier in the course of MVP from complex arrhythmias (Basso et al., 2015). Some risk factors have been proposed, including female sex, bileaflet prolapse, mitral annulus dilatation and disjunction, T wave inversion in the inferolateral leads, frequent and complex PVCs, and presence of papillary muscle fibrosis. By risk stratification, this patient is at a high risk of cardiovascular events such as heart failure and SCD. (Miller et al., 2018). Due to a lack of evidence, current guidelines, unfortunately, cannot provide specific indications for AMVP patients, and guidelines only recommend secondary prevention in the case of the non-surgery population as in our case. By applying sacubitril–valsartan, spironolactone, and bisoprolol, the condition of this patient is under good control. But the mass of melanoma progressed so fast that the complete removal of the tumor had no effect on this patient. In recent years, anti-PD-1 monotherapy is accepted as a first-line treatment for non–small–cell lung cancer (NSCLC) and other types of tumors and is approved by NCCN and EMSO (Hsu et al., 2019; Eguren-Santamaria et al., 2020; Leighl et al., 2021). Combined therapy with the anti-PD-1 antibody, including targeted therapy, immune agents, and chemotherapy has been tested in clinical trials or even in clinical practice (Davar et al., 2021; Janjigian et al., 2021). So under careful observation, the strategy of Toripalimab combined with temozolomide was applied to treat melanoma.

Surprisingly, after five courses of antitumor treatment, the patient presented good cardiac tolerance to our strategy. The total timeline of the medical process is shown in Table 2. There was no symptom of arrhythmia or other discomforts that appeared during the therapeutic process. There were no obvious changes observed from echocardiography (Table 3) and other auxiliary examinations, which means that the cardiac function was maintained in good condition. This case provides the first evidence of PD-1 combined with chemotherapy applied in the metastatic melanoma patient with AMVP. As we know, mitral valve prolapse (MVP) is a well-studied, mostly benign, phenomenon that can be caused by degenerative valve disease (mitral valve prolapse), left ventricular impairment and dilatation (in coronary artery disease or dilated cardiomyopathy), and infective endocarditis (Flachskampf and Daniel, 2006). In this case, we noticed that the patient not only suffered from MVP but also ventricular arrhythmia. According to a recent clinical study, though the pathophysiology of AMVP remains incompletely defined and uncertain, the outcome of AMVP was rarely severely observed by Holter monitoring. Most of the AMVP was independently associated with the phenotype dominated by mitral annulus disjunction, marked leaflet redundancy, and repolarization abnormalities (Essayagh et al., 2020). Considering that long-term arrhythmia results in notable excess mortality and reduced event-free survival, a well-designed study is necessary for further research. Toripalimab is proven to be safe to use for the AMVP patient without increasing the risk of heart failure or arrhythmia. We also confirmed the importance of standard prophylactic anti-CHF therapy, including angiotensin receptor neprilysin inhibitors (ARNIs), mineralocorticoid receptor antagonists (MRAs), and β-blockers during the treatment of antitumor (Edelmann et al., 2018). In conclusion, it is always a big challenge when a cardiologist faces the management and decision-making of a metastatic melanoma patient with basic cardiovascular diseases due to the lack of specific recommendations. The current case aimed at creating further awareness on reducing the cardiovascular risk through comprehensive therapy. Careful surveillance, prompt recognition, and appropriate treatment will become increasingly important. A close collaboration between cardiologists and oncologists is needed to improve the outcomes and survival of complex patients.

**TABLE 2:** Timeline of the medical process.

Time	Course of disease
October. 2019	Repeated gingival bleeding
December. 2019	Diagnosis of melanoma by pathological report
January. 2020	Cervical lymph node metastasis
AprIL. 2020	Toripalimab combined with temozolomide (1st course)
May 2020-June. 2020	Radiotherapy
July. 2020-August 2020	Toripalimab combined with temozolomide (2nd–5th course)

**TABLE 3:** Parameters of the echocardiography.

Parameter	Before the immunotherapy	After five-course of immunotherapy
AO	31 mm	34 mm
LA	42 mm	47 mm
LVSd	10 mm	9 mm
LVPWd	9 mm	8 mm
LVIDd	57 mm	64 mm
LVEF	57%	64%
RVIDd	26 mm	21 mm

AO, aorta; LA, left atrium; IVSd, interventricular septum dim; LVPWd, left ventricular posterior wall dimension; LVIDd, Left ventricular end diastolic dimension; LVEF, Left ventricular ejection fraction; RVIDd, Right ventricular end diastolic dimension.

## Data Availability

The original contributions presented in the study are included in the article Material. Further inquiries can be directed to the corresponding author.
